# Site-Specific N-Glycosylation on the AAV8 Capsid Protein

**DOI:** 10.3390/v10110644

**Published:** 2018-11-17

**Authors:** Arya Aloor, Junping Zhang, Ebtesam A. Gashash, Aishwarya Parameswaran, Matthew Chrzanowski, Cheng Ma, Yong Diao, Peng George Wang, Weidong Xiao

**Affiliations:** 1 Center for Diagnostics & Therapeutics and Department of Chemistry, Georgia State University, Atlanta, GA 30302, USA; aaloor1@student.gsu.edu (A.A.); egashash1@student.gsu.edu (E.A.G.); aparameswaran1@gsu.edu (A.P.); cma@gsu.edu (C.M.); 2 Sol Sherry Thrombosis Research Center, Lewis Katz, School of Medicine, Temple University, Philadelphia, PA 19140, USA; tug33619@temple.edu (J.Z.); tuh31047@temple.edu (M.C.); 3 School of Biomedical Science, Huaqiao University, Quanzhou 362021, China; diaoyong@hqu.edu.cn

**Keywords:** Adeno associated virus, mass spectrometry, site specific N-glycan analysis, host cell protein analysis, virus-host interaction

## Abstract

Adeno associated virus (AAV) is a versatile gene delivery tool, which has been approved as a human gene therapy vector for combating genetic diseases. AAV capsid proteins are the major components that determine the tissue specificity, immunogenicity and in vivo transduction performance of the vector. In this study, the AAV8 capsid glycosylation profile was systemically analyzed by peptide mass fingerprinting utilizing high-resolution mass spectrometry to determine the presence of capsid glycosylation. We identified N-glycosylation on the amino acid N499 of the capsid protein. We characterized the overall sugar profile for vector produced in 293 cells. Multiple N-glycosylated host-cell proteins (HCPs) copurified with AAV8 vectors and were identified by analyzing LC-MS data utilizing a human database and proteome discoverer search engine. The N-glycosylation analysis by MALDI-TOF MS, highlighted the probability of AAV8 interaction with terminal galactosylated N-glycans within the HCPs.

## 1. Introduction

Adeno associated virus (AAV) is a dependoparvovirus being used as a gene therapy vector for treating a variety of genetic disorders and acquired diseases. Lack of pathogenicity, low immunogenicity and differential tropism to multiple cell types make AAV a versatile gene delivery system [1]. This non-enveloped virus is approximately 25 nm in diameter and contains a unique linear single-stranded DNA genome [2,3,4]. AAV has a 4.8 kilobase genome flanked by two copies of 145 bp inverted terminal repeats [5]. AAV has two open reading frames (ORFs) consisting of the *Rep* and *Cap* genes. The Rep gene encodes four different replicating proteins (Rep78, Rep 68, Rep 52 and Rep 40) and the Cap gene encodes the three AAV8 capsid proteins (VPs) (VP1, VP2, and VP3) which are translated from different start codons at the same open reading frame (ORF) [2]. AAP has also been identified, and facilitates AAV packaging [6,7]. All three VPs are expressed in the same ORF but are created through alternative splicing maintaining a common C-terminal domain. VP1 contains an extra N-terminal sequence compared to VP2 and the VP2 contains an extra amino acid sequence at its N-terminal relative to VP3 [8]. Capsid proteins of AAV assemble to form a T1 icosahedral virion with sixty units of VP1, VP2 and VP3 in a ratio of 1:1:10 [9]. The structural studies of the intact capsid using cryo-electron microscopy, X-ray crystallography and image reconstructions revealed the N-terminal regions of VP1 and VP2 are folded inside the capsid structure [10], consequently blocking the N-terminal regions of VP1 and VP2 of binding activities. The common C-terminal VP3 region (~534 aa) determines the receptor binding of the virus. To date around 13 distinct serotypes of AAV have been used for gene therapy [11]. AAV serotypes display 55–99% sequence homology [2] but differ in their tissue tropism [12,13,14]. Amino acids of a particular serotype determine its structural dynamics and tissue specificity [15].

Up until now most work in the field has focused on AAV2, but further insight into the other serotypes is needed to obtain a greater understanding of AAV. AAV8 is widely known for its high performance in liver transduction, and is the preferred vector targeting the liver in gene therapy [5,9,16,17,18]. AAV8 is utilized in treatment research for the hemophilia A, hemophilia B, familial hypercholesterolemia and glycogen storage disease type II [16,18,19,20,21,22,23]. AAV8 has been documented transducing cardiac and skeletal muscle in hamster and mice [24].

Host cell glycosylation plays a significant role in AAV viral entry, tissue selection, and infectivity. These roles are partially documented by identified AAV receptors, which are often glycans. Heparin sulphate, N-glycans terminated with galactose, and sialic acid are well-known primary receptors of various AAV serotypes [25,26,27]. The secondary receptor of AAV8 is a laminin receptor (LamR), a host cell surface glycoprotein [17]. Some AAV serotypes have notable differences in terms of in vivo performances and secretion efficiency which are characteristically features that can be influenced by glycosylation state. Here we utilized high-resolution mass spectrometry with intensive sample preparation to explore potential glycosylation in AAV8 capsid protein.

Typically in non-enveloped viruses glycosylation is less common, but the capsid protein of hepatitis E and fiber protein of adenovirus-5 are glycosylated [28,29]. Post translational modifications of capsid proteins have a drastic impact on viral properties particularly regarding tropism. Glycosylation may also affect the immunogenicity of viruses if it is part of the capsid components. Site specific modifications of capsid proteins like tyrosine phosphorylation is reported to promote ubiquitination and degradation of AAV2 capsid protein leading to decreased tropism [30]. Similarly, AAV tropism may be influenced by the presence of glycosylation on the capsid protein. To date AAV has widely been deemed a non-glycosylated DNA virus. Here we presented direct evidence of N-glycosylation on the NNS_499–501_ AAV8.

## 2. Materials and Methods

### 2.1. Source of Virus Sample

The AAV8 vector was expressed using HEK 293 cell line and purified by density gradient centrifugation [31]. Both secreted and intracellular AAV8 were purified from the same AAV batch using differential purification at Sol Sherry Thrombosis Research Center (Temple University, Philadelphia, PA, USA).

### 2.2. Viral Protein Sample Preparation

The samples were concentrated using a speed vac to minimize the water content, and the proteins were precipitated using ice-cold ethanol (Decon Labs, PA 19406). The protein pellet was dissolved in urea buffer (6 M urea in 0.1 M Tris/HCl, pH 8.5) and the concentration of the protein mixture was calculated using BCA protein detection kit (Thermo Fisher Scientific, Waltham, MA, USA) according to the kit protocol using urea buffer as a blank. The protein mixture was denaturated by adding 1 M Dithiothretiol, (DTT, Acros Organics, Morris Plains, NJ, USA) in 100:1 *v*/*v* ratio to the reaction mixture and heated at 95 °C for five minutes then cooled and added 1 M iodoacetamide (IAM, from Acros Organics, Morris Plains, NJ, USA) in the ratio of 50:1, *v*/*v* at 37 °C for 1 h (reductive alkylation reagents were purchased from Acros Organics, Morris Plains, NJ, USA). The reduced and alkylated samples were desalted, and buffer exchanged with 50 mM ammonium bicarbonate, pH 8.0 buffer, using Microcon-10 kDa (YM-10, 0.5 mL, Millipore, Burlington, MA, USA) and aliquoted to three parts.

All other reagents used here, which are not categorically mentioned were additionally purchased from Sigma-Aldrich (St. Louis, MO, USA).

### 2.3. SDS–PAGE and Visualization of Glycoprotein Band and In-Gel-Digestion

The capsid proteins of AAV8 particles were separated (around 10 µg of protein) on a 4–12% Bis-tris gel by SDS-PAGE. (30% acrylamide-bis acrylamide solution was purchased from BioRad, Herculus, CA, USA, 94547) The glycoprotein detection kit (Thermo Fisher Scientific, Waltham, MA, USA) was utilized to stain the glycoprotein band segregated on the SDS-PAGE gel. The glycoprotein staining was performed according to the manufacturer’s protocol. In principle, the protein gel is treated with periodic acid (oxidizing reagent), glycols present in the sugar moieties of glycoproteins are oxidized to aldehydes. During this reaction a magenta pink band develops wherever the glycans are present, while the rest of the protein remains invisible. The gel was further stained by Coomassie blue to visualize the protein bands. To perform the in-gel digestion and further mass spectrometric analysis, a parallel gel was prepared and the stained exclusively with Coomassie blue. The corresponding bands identified from glycoprotein staining were excised for in-gel digestion referred to pre-established protocol [32,33]. The 10 µg of PNGase F (New England Biolabs, Ipswich, MA, USA) treated samples were loaded adjacently with the same amount of control samples to compare the protein profile and band shift after de-N-glycosylation.

### 2.4. N-Glycan Analysis

Intracellular and secreted AAV8 (~100 µg each) were treated with ~50 Units of PNGase F. The sample was incubated at 37 ° C for 16 h. The N-glycans released were separated by ethanol precipitation. Precipitated proteins were separated by centrifugation and the supernatant contained the released glycan. The N-glycans were dried using vacufuge and tagged with anthranilic acid (2 amino benzoic acid, 2AA) according to the previously published protocol [34,35]. An excess of labelling agent was removed by ethyl acetate wash. The 2AA-labelled N-glycans were reconstituted in MS grade water and drop dialyzed on nitrocellulose membrane filter 0.05 µm VMWP (Millipore, Burlington, MA, USA) in water for 30 min. The dialyzed samples were concentrated to 1 µL in a Speed-Vac Vac and commixed with 1 µL of a saturated solution of Dihydroxy benzoic acid (DHB) matrix prepared in 70% Acetonitrile (ACN) in water (Mass spec grade ACN, J.T Baker chemicals, Avantor Performance Materials, Inc., Center Valley, PA, USA) A 1 µL of the sample was spotted on the MALDI- plate and allowed to dry and form crystals. The plate was installed in the instrument, and the sample spot was bombarded with 32.5% high energy laser power utilizing a MALDI-TOF-MS (Ultrafle Xtreme, Bruker Daltonics; Bremen, Germany) system to acquire all the MS spectra. The data acquisition was in negative ionization using reflectron mode. The spectra were generated with the uniform signal intensity. The glycoworkbench (http://code.google.com/p/glycoworkbench/) was availed to annotate the *m*/*z* values in the spectra and give the structural identification to the human system. The MS/MS spectra of the major glycoforms, confirmed the structures. The tandem mass spectrometry was performed using “LIFT” mode (negative ionization) bombarded with high energy laser. The pattern of fragmentation was attesting the structure and composition of each oligosaccharide moiety. The relative intensity of the glycan masses calculated by Flex Analysis software (Bruker Daltonics, Billerica, MA, USA) to produce the final spectra.

### 2.5. Peptide Mapping Analysis

The third aliquot of the sample around 100 µg was treated with sequencing grade trypsin (Promega, Madison, WI, USA) in an enzyme to protein ratio of 1:50 (*w*/*w*) and incubated at 37 °C for 16 h. The reaction was stopped by heating the tube in a boiling water bath for five minutes. Approximately 50 µg of the peptide was saved for further analysis and the other aliquot was digested with Glu C (Promega, Madison, WI, USA) in an enzyme protein ratio of 1:50 (*w*/*w*) and incubated at 37 °C overnight. The resulted peptides were then subjected to glycosylated peptide enrichment, higher-energy C-trap dissociation (HCD) analysis, N-glycosite occupancy, and protein identification.

### 2.6. Glycosylated Peptide Enrichment

The Click Mal, a HILIC media (ACCHROM, Beijing, China) was used to enrich the glycosylated peptide from the virus sample according to a previously published method, with minute modifications [36]. An in-house HILIC-SPE column was prepared by inserting a small C8 disc (Empore C8 disk, Bioanalytical Technologies, St. Paul, MN, USA) into a 200 μL tip. Around 6 mg of HILIC media was weighed out and washed with 100 µL of neat ACN and then transferred to the prepared microtip to make the HILIC-SPE column. The column was then washed by passing with 100 μL of 10% ACN containing 0.1% FA using a microsyringe and then equilibrated with binding buffer (BB; 80% ACN containing 1.0% FA) for 3–5 times. The proteolytically (trypsin and trypsin followed by Glu C) digested peptides were dried and resuspended in 10 μL of binding buffer and introduced to the microcolumn and allowed to bind to the column for 10 min at room temperature. The unbounded peptides were washed off by passing 100 µL of binding buffer and repeated the step for five times. The column bound glycosylated peptide were then eluted out utilizing 200 μL of the elution buffer (EB; 1.0% FA in Water). The HILIC-enriched glycosylated peptide were then dried using speed vac and resuspended in 5 μL of 2% ACN containing 0.1% FA in H_2_O and injected to the nano-LC orbitrap MS-system.

### 2.7. N-Glycosite Detection (^18^O Labelling)

A fraction of (approximately 40 µg) proteolytic enzymes treated intact peptides were subjected to glycosylation site identification. The digest was thoroughly dried in a speed Vac and resuspended in 10 µL of ABC buffer (ammonium bicarbonate prepared in H_2_^18^O, pH 8.0). The enriched glycosylated peptides were treated with 1 µL of PNGase F (50 U), and the mixture was incubated at 37 °C for 16 h.

### 2.8. Peptide Analysis by LC-MS/MS

The intact, enriched and ^18^O labelled peptides were dried and resolved in 2% ACN containing 0.1% FA. 5 μL of the intact glycosylated peptides or 3 μL of the ^18^O-labeled N-deglycosylated peptides were injected to Dionex Ultimate 3000 RSLC nano System (Thermo Fisher, Waltham, MA, USA). The LC system has a Nano Trap column, (Acclaim Pep Map100 C18 (2 cm × 75 μm I.D, 3 μm)). The flow rate was adjusted to 5 μL/min with mobile phase A (2% ACN, 0.1% FA) for 10 min for sample trapping, and separated on the Easy-spray Pep Map C18 Column (15 cm × 75 μm I.D., 3 μm, 100 Å). The separation was accomplished by a 120 min linear gradient (3 to 40% Mobile Phase B (80% ACN, 0.1% FA) at the flow rate of 300 nL/min. The column was washed for 10 min with 99% B and reconditioned with 1.0% B for 5 min for prior to the next run [37].

An elite mass spectrometer with the spray source (1.6 kV) of (Thermo Fisher) LTQ-Orbitrap is integrated with the LC system. The LTQ Orbitrap mass spectrometer was adjusted to data-dependent mode with an alternating MS1 and MS 2 acquisition. The Orbitrap mass analyzer MS scan was performed with the mass range, *m*/*z* 400–1600; resolution at *m*/*z* 400, 6 × 104; automatic gain control target (AGC), 106 ions and maximum ion accumulation time, 50 ms. The MS1 ions of the ten most intense species were subjected to MS/MS collision-induced dissociation (CID) in the ion trap analyzer. The MS/MS scan model was performed in centroid scan model. CID-MS parameters were set by giving default charge state as 3, activation Q was 0.25 with an activation time 5.0 ms and isolation width was set to *m*/*z* 3.0. The normalized collision energy was set up to 35%.

For the HCD mode, orbitrap analyzer was set at a resolution of 15,000 at *m*/*z* 400; AGC was 10,000 ions, and maximum ion accumulation time was increased to 200 ms. All the CID set parameters remained the same except the parameters of activation time which set up to 0.5 ms and the isolation width of *m*/*z* 2.0. The /MS parameter was performed at 27% NCE. For MS/MS data interpretation for LC−CID−MS data analysis of deglycosylated peptides, pFind software 2.8 (http://pfind.ict.ac.cn) was used [36,37,38]. FASTA sequence of AAV8 sequence were created using the already published sequences of VP1, VP2 and VP3 [9]. Tolerance of peptide mass was set to 20 ppm, and the fragment ion tolerance was 0.5 Da. Since the proteins were reduced and alkylated, the fixed modification was carboxyamidomethylation of cysteine (+57.021 Da), and the variable modifications were set to detect the deamidation of N and Q (+0.984 Da) and oxidation of methionine (+15.995 Da). Two maximum missed cleavage sites were selected for trypsin (KR-C) and Glu C (DE). For the glycosite identification: CID−MS/MS data analysis of ^18^O- labelled de-N-glycosylated peptides were performed by setting the precursor ion mass between 350 and 6000 Da. Along with above described search parameters, we have included N-deamidated with ^18^O (+2.988 Da) and specified as variable modifications with FDR < 0.1. The theoretical glycosylated peptide masses were obtained from the online server protein ExPAsy (http://web.expasy.org/peptide_mass) and GlycoMod tool (http://web.expasy. org/glycomod) possibility of glycosylations and sites of glycosylation by consensus sequence predicted by NetNglyc (http://www.cbs.dtu.dk/services/NetNGlyc) were further related manually to the data which we obtained from the independent N-glycan analysis [39].

### 2.9. HCP Identification by LC-MS/MS Analysis

In the MS analysis of the peptide mapping, data were sanctioned to detect the host-cell protein (HCP) glycoprotein present in the AAV 8 sample. The multiple protein identification in the sample was performed by Proteome Discoverer^TM^ (Thermo Fischer Scientific). Human database was downloaded from UniProt (2016_02 Release, 20,198 reviewed entries) which can also detect even the common process-induced contaminant proteins, like keratin. The database was edited by adding AAV8 VP3 which is the most abundant protein in the sample. AAV 8 VP sequence was taken from a previously published article, and FASTA files were generated and uploaded to the search engine [36,40,41]. The database was used to identify the other proteins apart from AAV8 in the sample especially host cell proteins (HCP) which is interacted to AAV 8. The precursor mass tolerance was set to 20 ppm and fragment mass tolerance was set to 0.8 Da. The data was generated from trypsin digested sample hence the enzyme entered used for the given search was trypsin. All the possible dynamic modifications, like glutamine pyroglutamine conversion on any N- terminus(−17.027Da), methionine oxidation (+15.995 Da), acetylation (K, +42.011 Da) and deamidation (N, Q/+0.984) were considered in the specification. Since we have performed ^18^O labelling, we included specific deamidation (N/+2.988 Da) also. Static modification is set to carbamidomethyl (C/+57.021 Da). A strict target false discovery rate was set to 0.01–0.05.

## 3. Results

**Figure 1:**
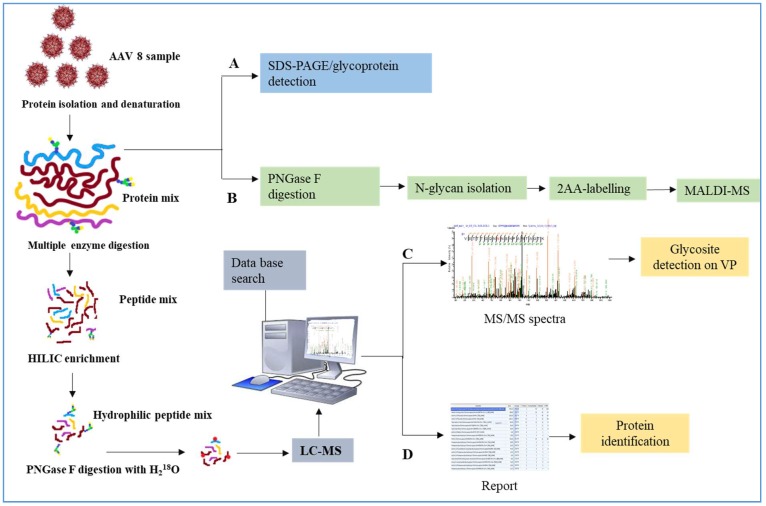
Intracellular or secreted Adeno associated virus (AAV)8 were processed in parallel and analyzed using multiple techniques. (**A**) Samples visualized on the SDS-PAGE gel by glycoprotein labelling. (**B**) N-glycan analysis using MALDI-MS identifies the type of glycosylation. The peptides generated by Glu C or trypsin digestion were enriched using HILIC cartridges. Deglycosylation of enriched glycosylated peptide in the presence of H_2_^18^O labelled the glycosite with ^18^O. (**C**) The samples were injected to LC-MS and using the database of AAV8 VPs, the N-glycosite was identified (**D**) The same data was searched against the human database to discover which proteins were copurified with AAV8.

AAV has a complicated capsid structure consisting of multiple units of VP1, VP2 and VP3. Figure 1 details our methodology for characterizing AAV capsid glycosylation. SDS-PAGE was used to detect the protein profile of the virus and the presence of any host cell proteins, copurified. Additional glycoprotein staining techniques along with N-glycan analysis using MALDI-MS and LC-MS were used. Both HCP and AAV capsid glycosylation were investigated.

### 3.1. Detection of Glycosylation by SDS-PAGE

**Figure 2:**
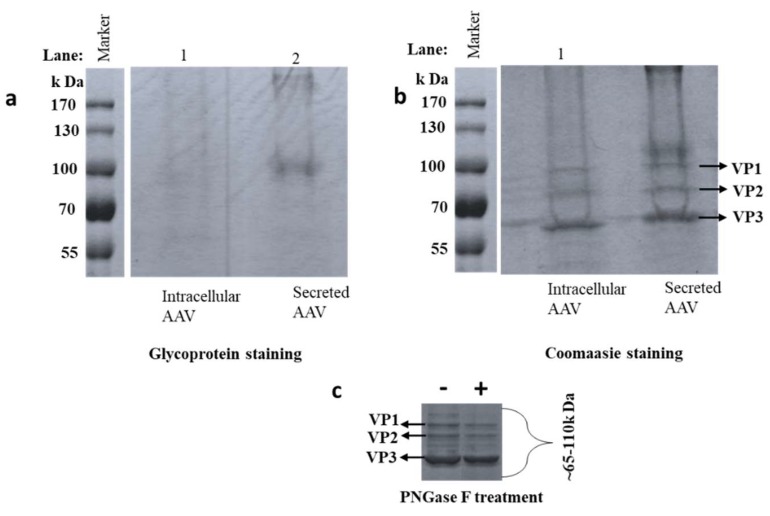
AAV8 Glycosylation detection. AAV8 derived from the cell (intracellular), and media (secreted) are separated on SDS-PAGE (**a**) protein bands visualized after glycoprotein staining. Glycosylated bands were observed near the AAV8 capsid protein (VP) region (55–100 kDa). Gel (**b**) is gel (**a**) stained with Coomassie brilliant blue to visualize the whole protein profile. (**c**) Independent Coomassie stained gel run after de-N- glycosylation of secreted sample. (+ and − the addition of PNGase F).

AAV8 samples were denatured and separated on 12% SDS-PAGE. VP1, VP2, and VP3 were segregated in the region of 50–100 kDa determined by the protein marker [42]. The capsid proteins VP1, VP2, and VP3 have molecular weights around 87 kDa, 73 kDa and 60 kDa, respectively [15]. Faint magenta pink colored bands in the VP regions were identified, suggesting VP glycosylation (Figure 2a). A dark magenta pink band was observed around 100 kDa in the AAV8 secreted sample, suggesting the presence of host cell glycoprotein in the sample. Non-VP bands (~200kDa, and 110 kDa) may be an indicator of glycosylated HCP in the sample. Gel staining by Coomassie brilliant blue visualized the total protein profile on the SDS-PAGE (Figure 2b). Glycosylated bands between the VPs vanished after PNGase F treatment suggesting that these bands were N-glycosylated VPs (Figure 2C). The difference in glycoprotein bands from intracellular and secreted AAV8 may signify the different intermolecular interactions of VPs with various glycoprotein which may copurify with AAV particle in the purification procedures. Host cell proteins can copurify along with the VPs if they have a similar molecular weight, which may lead to ambiguity in the analysis. Some copurified proteins are found to be heavily glycosylated and stained positively by glycoprotein staining. This technique was considered only for preliminary examination, and peptide mapping designates protein identity of the HCPs by high-resolution LC-MS with the aid of database search.

### 3.2. The N-Glycan Profile of Intracellular Derived AAV8 and Media Derived AAV8

**Figure 3:**
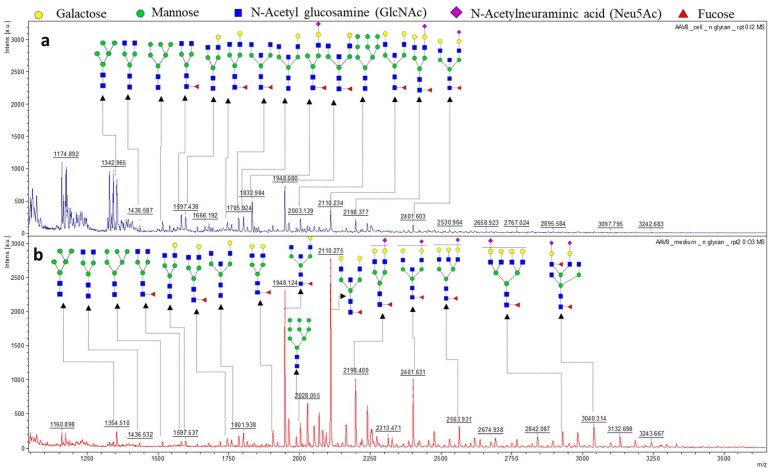
MALDI- MS spectra of 2-AA derived N-glycan in negative mode [M−H]^−^. (**a**) The whole glycome profile of intracellular AAV8. (**b**) The whole glycome analysis of secreted AAV8. The most intense peaks from both the spectra (*m*/*z* 2110.234, 1948.088, 2401.633, 1832.984 were further confirmed by MS/MS fragmentation spectra in MALDI-TOF MS in lift mode (supplemental Figure S2).

The intracellular and secreted AAV8 samples were both treated with PNGase F in preparation for N-glycome analysis. The N-glycans of corresponding samples were isolated and tagged with 2 Aminobenzoic acid (2-AA) then spotted on MALDI-plate to provide unique N-glycan profiles of the corresponding samples. The resulting spectra were smoothed, and the baseline subtracted. The MS/MS of high-intensity peaks were performed in “Lift mode” to confirm the monosaccharide composition. The peak annotation was done using the Glycoworkbench software following verification by the CFG database (Figure 3). Since the protein identification data suggested the presence of host cell glycoproteins in the samples, the N-glycosylation profile of the secreted and intracellular AAV8 cannot be directly linked to the AAV8 capsid protein (VP). However, the high intensity of secreted AAV8 glycan spectra correlates with the glycoprotein detection data and suggests the possibility of AAV8 interaction with host cell glycans. The majority of the N-glycans identified in the sample were terminally galactosylated, indicating the capsid protein’s affinity to the terminally galactosylated HCPs. The glycoforms are identical in structure in both samples (Table S1). The difference in proportion and intensity of each glycoform is noticeable (Figure 3).

### 3.3. The Glycosylation Analysis at Peptide Level

**Figure 4:**
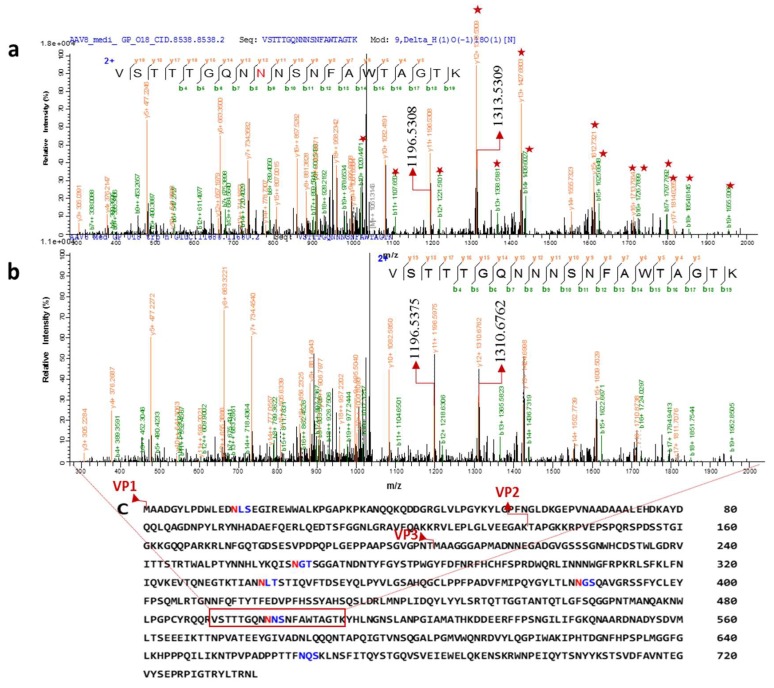
Two distinct tandem mass spectra of peptide sequence “VSTTTGQNNNSNFAWTAGTK” of the AAV8 capsid protein (in the common region of VPs). Characterized CID spectra of glycosylated peptide after ^18^O mediated digestion. (**a**) The position of de-N-glycosylated asparagine 499 (^18^O-incorporated aspartic acid) was attested by mass increment of 2.8547 Da (theoretical mass difference 2.98 Da) to the series of b and y ion (the precursor ion mass: m/z 2101.93193). (**b**) The position of Non-glycosylated asparagine 499 in the sequence was confirmed by y12/b8 ions (the precursor ion mass: *m*/*z* 2099.0766 Da). The highlighted ions (marked with a star) shows the mass differences from the non-glycosylated peptide mass spectrum. y11+ (highlighted) ions are same in both the spectra. (**c**) Capsid protein amino acid sequence and possible N-glycosylation sites predicted by NetNglyc software based on the consensus sequence (NXT/S, “X” can be any amino acid except proline). Different N-terminal sequence of VP1, VP2 and VP3 are marked in the sequence. The glycosite identified peptide is highlighted on the sequence.

To obtain peptide mass fingerprinting data, samples were prepared by in-solution digestion. Peptides were run on Orbitrap to obtain the peptide mass fingerprinting data. Consensus sequence search of N- glycosylation NXT/S through GlycoMod revealed 6 possible glycosites; N_14_LS, N_263_GT, N_338_LT, N_385_GS, N_499_NS, and N_665_QS (Figure 4C). Aside from N_14_, every other site is in the common region of VPs. N_14_, is in the N-terminal domain and looped inside the capsid assembly [10]. Thus, the analysis of glycosylated peptide focused on VP3 sequence due to the presence of all predicted glycosylation sites and abundance of peptide in the total sample.

To characterize the N-glycosylation site of capsid proteins, we focused on HCD mass spectra of tryp/Glu C digested proteins of intracellular and secreted AAV8. The peptides indicated N-glycosylation on multiple peptides at different retention times with the marker fragment ions in MS2 spectra (oxonium ions at *m*/*z* 366 (HexHexNAc1,1 +), *m*/*z* 292 (Neu5Ac, 1+), *m*/*z* 204 (HexNAc, 1+), *m*/*z* 162 (Hex, 1+), and sub fragment ions at *m*/*z* 186 (HexNAc- H_2_O, 1+), *m*/*z* 168 (HexNAc-2H_2_O, 1+), *m*/*z* 138 (HexNAc-2 H_2_O−CH3OH, 1+), *m*/*z* 126 (HexNAc-2H_2_O−CH3COH, 1+), and *m*/*z* 274 (Neu5Ac− H_2_O, 1+)). To enhance the intensity of detected glycosylated peptides, which is covalently linked to multiple glycoforms, the HILIC-enriched glycosylated peptides were de-glycosylated by PNGase F in the presence of ^18^O water. Thus, the weak signals generated from scattering the peptides over the reverse phase column because of differential compositions of glycoforms covalently linked to the same peptide are eliminated. Furthermore, the signals from peptides derived from host cell proteins (HCPs) were hindering the glycosylated peptide signals. We then switched to CID analysis of the de-glycosylated peptides, which have an equal chance of vapor ionization in the mass analyzer as non-glycosylated peptides. A shift of 2.988 Da (^18^O -deamidated Asn) in the MS spectrum indicates a glycosylation site. De-N-glycosylated asparagine 499(^18^O-incorporated aspartic acid) witnessed a mass increment of 2.8547 Da (theoretical mass difference 2.98 Da) indicating that N_499_ is glycosylated (Figure 4A). The fragment ions starred in the Figure 4A show the mass differences from the spectra corresponding to non-glycosylated peptide. The experiment was repeated with another batch of AAV8 and CID spectra showed a reproducible spectrum with the mass difference of 3.03 Da from y^12+^-y^17+^ ions in the MS/MS spectra (Figure S1).

Glycosylation on the capsid protein at N_499_ was identified and verified by MS/MS analysis. Since the amount of glycosylated peptide was below the limit of detection (LOD), in normal enzymatic digest (trypsin/Glu C and trypsin alone) analysis, trypsin and Glu C trials failed to pick up the glycosylated peptides. The glycosylated peptide was identified from media isolated AAV8 sample, only after HILIC enrichment. The entire experiment was repeated with an independent batch of AAV8 and reconfirmed the result (Figure S1). The samples showed the presence of both glycosylated and non-glycosylated peptide. As enrichment process may cause the loss of non-glycosylated peptide, we could not calculate the percentage of glycosylation.

**Figure 5:**
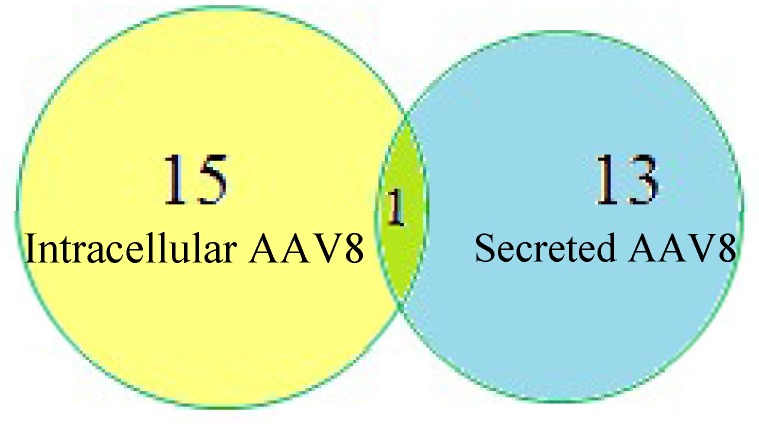
Host-cell protein (HCP) identification in intracellular and secreted AAV8 using Proteome Discoverer^1.4^.

We identified various host cell glycoproteins in the sample that copurified along with AAV8. Proteins isolated from the media and cells were analyzed using proteome discover^1.4^ software. The search results are listed in Table 1. We included only the proteins with greater than 20% coverage. We identified fifteen HCPs in the intracellular AAV8 sample and thirteen HCPs in the secreted AAV8. Galactose 3 binding protein(G3BP) is the only prevalent protein identified in both the samples (Figure 5). Many of the proteins identified by the data search are known to be heavily N-glycosylated.

## 4. Discussion

Study of the post translational modifications of AAV8 is an underserved area. Our study identified the presence of N-linked glycans in purified samples of AAV8. N-glycome analysis of intracellular and secreted vectors confirmed the presence of N-glycosylation in the overall viral sample indicating interactions between AAV and glycosylated proteins. The N-glycosylation pattern in terms of proportion and MS profile is unique for secreted and intracellular virus and showed the presence of a high amount of N-glycoforms. The glycoform analysis indicates that both AAV samples contain highly galactosylated species as the major sugar. Heparin sulphate, N-glycans terminated with galactose, and sialic acid are the well-known primary receptors of various AAV serotypes [11]. The identification of glycosylated proteins interacting with AAV8 is interesting given AAV’s known predilection for glycosylated proteins [26,43,44,45] While some of these proteins could be due to issues with purification steps it does beg for further research into the interactions that AAV has with glycans in the cell and their importance in secretion of AAV. The close interaction of AAV8 with different host cell glycoprotein also suggests an additional clue about viral tissue tropism.

AAV serotypes have notable differences in terms of in vivo performances and secretion efficiency, which are characteristic features that may have be influenced by the glycosylation state of AAV. We studied different aspects of N-glycosylation in AAV8 recombinant vector and confirmed the presence of N-glycans on N_499_ on “VSTTTGQNNNSNFAWTAGTK” peptide, localized on the common region of the AAV8 capsid protein. A previous study had investigated AAV2 glycosylation and failed to identify any glycosylation events on the capsid [42]. The glycosylated peptide and non-glycosylated variant coexisting in the AAV preparations suggests the existence of multiple pathways for AAV8 maturation post AAV packaging. We hope with further study to determine how exactly a part of the population comes to be glycosylated while the rest is not. Also important are the possible downstream effects with regards to yield and transduction efficiency. It will be important to study how glycosylation of AAV and its copurified proteins affect neutralizing AAV antibody and in vivo performance.

## 5. Conclusions

Adeno associated virus vector is an important gene delivery tool for both basic scientific research and human gene therapy. This study mainly focused on elucidating the capsid protein structure profile arising from post translational modifications, primarily the glycosylation. Virus capsid glycosylation is a potential posttranslational modification which may affect tissue tropism, vector immunogenicity and vector intra cellular trafficking. Here we identified the existence of natural N-glycosylation on the AAV8 capsid protein. The chemical details of capsid glycosylation are revealed. These results provide new insights into the AAV vector production process, as well new pathways for future vector capsid enhancement.

## Figures and Tables

**Table 1:** HCP identification using Proteome Discoverer^1.4^.

UniProt Accession No.	Protein Identity	Coverage%	
		Intracellular	Secreted
P0DP23	Calmodulin 1 (Human)	38.93	ND
O60637-3	Tetraspanin-3 (Human)	34.39	ND
P06748-3	Nucleophosmin (Human)	34.36	ND
Q92542-2	Nicastrin (Human)	33.53	ND
Q9BY67-2	Cell adhesion molecule 1 (Human)	33.33	ND
P13473	Lysosome-associated membrane glycoprotein 2 (Human)	32.44	ND
Q5ZPR3-3	Isoform 3 of CD276 (Human)	29.61	ND
P11279	Lysosome-associated membrane glycoprotein 1 (Human)	29.5	ND
Q14108	Lysosome membrane protein 2 (Human)	28.24	ND
Q13740	CD166 (Human)	27.1	ND
Q6PCB8-2	Embigin (Human)	25.27	ND
Q08380	Galectin-3-binding protein (Human)	23.93	36.75
P60900	Proteasome subunit alpha type-6 (Human)	22.76	ND
P05556-2	Isoform 2 of Integrin beta-1 (Human)	22.18	ND
Q13162	Peroxiredoxin-4 (Human)	21.03	ND
P12268	Inosine-5′-monophosphate dehydrogenase 2 (Human)	ND	19.65
P68363-2	Tubulin alpha-1B chain	ND	41.49
P01834	Ig kappa chain C region (Human)	ND	35.85
P02751-5	Isoform 5 of Fibronectin (Human)	ND	33.7
P01619	Ig kappa chain V-III region (Human)	ND	31.19
P23142-4	Isoform C of Fibulin-1	ND	30.75
P23142	Fibulin-1	ND	30.3
Q16222-2	Isoform AGX1 of UDP-N-acetyl hexosamine pyro phosphorylase	ND	29.31
P07437	Tubulin beta chain	ND	26.35
P0CG48	Polyubiquitin-C	ND	23.65
Q04837	Single-stranded DNA-binding protein, mitochondrial	ND	22.3
P35556	Fibrillin-2 (Human)	ND	20.81

Note: ND—Not detected.

## Supplementary Materials

Supplementary materials can be found at http://www.mdpi.com/1999-4915/10/11/644/s1, Figure S1: Reconfirmation of N-glycosite on NNS499-501, Figure S2: Tandem mass spectrometry of dominant 2AA labelled glycoforms in Lift mode, Table S1: Glycoforms identified in differentially purified AAV8 sample.
