# Supplementary material for: Site-Specific N-Glycosylation on the AAV8 Capsid Protein
Source: Viruses. 2018 Nov 17;10(11):644. doi: 10.3390/v10110644 (PMC6266768; doi:10.3390/v10110644)
Supplement: Supplementary file 1 [file viruses-10-00644-s001.pdf]

Supplementary data

Arya Aloor[1#], Junping Zhang[2#], Ebtesam A. Gashash[1], Aishwarya Parameshwaran[1], Matthew Chrzanowski[2], Cheng Ma[1], Yong Diao[3], Peng George Wang[1]*, Weidong Xiao[2]*.

[1]Center for Diagnostics & Therapeutics and Department of Chemistry, Georgia State University, Atlanta, Georgia 30302, United States

[2] Sol Sherry Thrombosis Research Center, Lewis Katz, School of Medicine, Temple University, Philadelphia, Pennsylvania 19140, United States

[3] School of Biomedical Science, Huaqiao University, Quanzhou, China

Table of contents

Figure S1: Reconfirmation of N-glycosite on $NNS_{499\text{-}501}$ S1

Figure S2: Tandem mass spectrometry of dominant 2AA labelled glycoforms in Lift mode S2

Table S1: Glycoforms identified in differentially purified AAV8 sample S3

Figure S1: Reconfirmation of N-glycosite on $NNS_{499-501}$

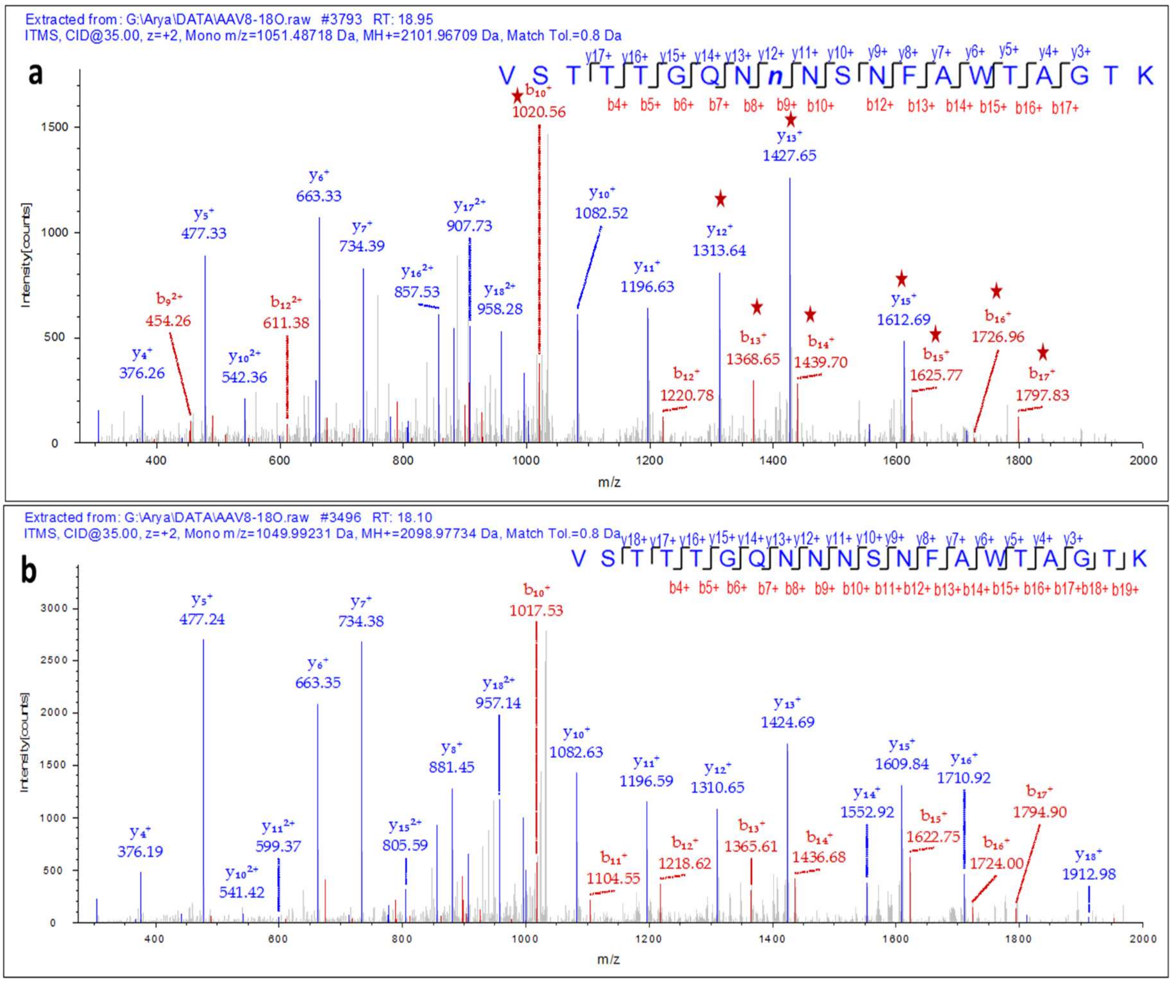

Figure S1. Reconfirmation of N-glycosite identification. The AAV8 batch is expressed purified, processed and analysed independently from the previous analysis. a) CID spectra of the ^{18}O labelled glycopeptide b) CID spectra of nonglycosylated peptide in a single run.

Figure S2: Tandem mass spectrometry of dominant 2AA labelled glycoforms in Lift mode

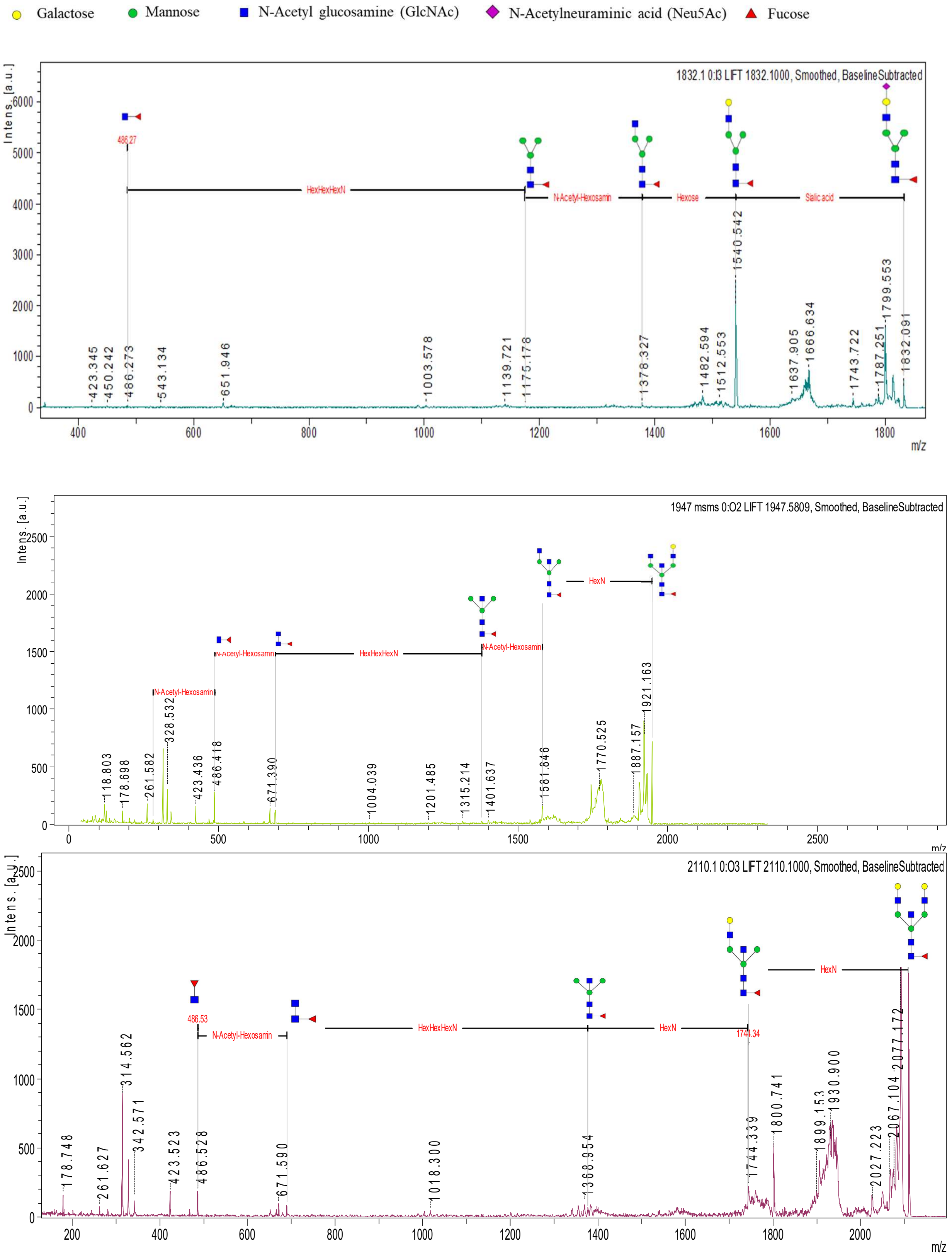

Table S1. Detected N-glycoforms associated to secreted and intracellular AAV8 samples. The Major glycoforms are confirmed by MS/MS and minor species are identified by GlycoWorkbench and CFG database search.

○ Galactose ● Mannose ■ N-Acetyl glucosamine (GlcNAc) ◆ N-Acetylneuraminic acid (Neu5Ac) ▲ Fucose

	Experimental m/z		Theoretical m/z)	Structure	Composition	Type
No	Secreted	Intracellular				
1	1354.510	1354.458	1354.4789	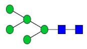	H5N2	High mannose
2	1436.532	1436.587	1436.532	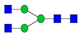	H3N4	Complex
3	1516.606	1516.618	1516.5317	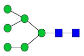	H6N2	High mannose
4	1541.654	1541.576	1541.5631	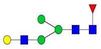	H4N3F1	Complex
5	1558.431	1557.538	1557.5873	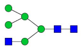	H5N3	Hybrid
6	1582.710	1582.658	1582.5872	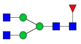	H3N4F1	Complex
7	1597.537	1597.438	1598.5848	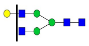	H4N4	Complex
8	1678.516	1679.583	1678.5846	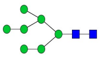	H7N2	High mannose
9	ND	1686.738	1686.6009	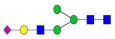	H4N3S1	complex
10	1719.742	ND	1719.611	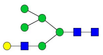	H6N3	Hybrid
11	1745.000	1744.814	1744.6427	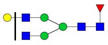	H3N4F1	Complex
12	1760.959	1760.903	1760.6377	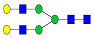	H4N4	Complex
13	1785.973	1785.924	1785.669	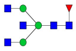	H3N5F1	Complex
14	1801.938	1801.882	1801.6642	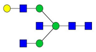	H4N5	Complex
15	1833.035	1832.984	1832.5688	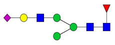	H4N3S1F1	Complex

16	1841.019	1841.849	1840.6304	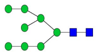	H8N2	High mannose
17	1848.866	1848.956	1848.6537	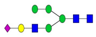	H5N3S1	Hybrid
18	1865.875	ND	1865.669	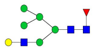	H6N3F1	Hybrid
19	1881.924	ND	1881.664	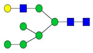	H7N3	Hybrid
20	1890.984	ND	1889.680	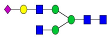	H4N4F1	Complex
21	1907.067	1907.089	1906.696	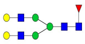	H5N4F1	Complex
22	1948.124	1948.080	1947.722	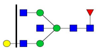	H4N5F1	Complex
23	1989.172	1989.183	1988.749	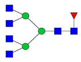	H3N6F1	Complex
24	2003.070	2003.139	2002.690	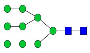	H9N2	High mannose
25	2028.055	2027.958	2027.722	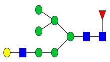	H7N3F1	Hybrid
26	2052.218	2053.157	2051.733	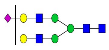	H5N4S1	Complex
27	2069.126	2069.191	2068.748	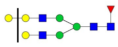	H6N4F1	complex
28	2092.760	2094.338	2092.760	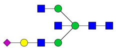	H4N5S1	Complex
29	2110.275	2110.234	2109.775	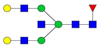	H5N5F1	Complex
30	2198.400	2198.377	2197.791	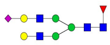	H5N4S1F1	complex
31	2313.471	2313.569	2312.854	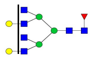	H5N6F1	complex
32	2401.631	2401.603	2400.870	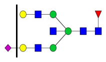	H5N5S1F1	Complex
33	2475.764	2475.78	2474.907	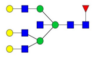	H6N6F1	Complex
34	2489.599	2489.867	2488.886	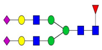	H5N4S2F1	Complex
35	2530.676	2530.964	2529.913	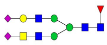	H4N5S2F1	Complex
36	2563.931	2563.828	2562.923	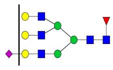	H6N5S1F1	Complex
37	2604.750	2604.881	2603.950	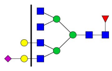	H5N6S1F1	complex

38	2691.961	2693.006	2691.966	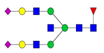	H5N5S2F1	Complex
39	2750.676	ND	2748.987	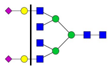	H5N6S2	Complex
40	2767.100	2767.024	2766.002	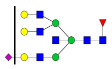	H6N6F1S1	Complex
41	2842.087	ND	2840.039	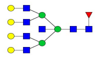	H7N7F1	Complex
42	2894.984	2895.584	2895.045	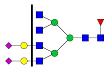	H5N6S2F1	Complex
43	2930.047	2930.072	2928.055	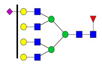	H7N6S1F1	Complex
44	3098.125	3097.795	3098.124	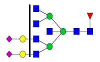	H5N7S2F1	Complex
45	3132.698	3132.202	3131.132	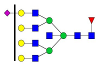	H7N7S1F1	Complex
46	3185.324	3186.702	3186.141	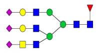	H5N6S3F1	Complex
